# Closing gaps, opening doors: an experimental collaboration in stem cell intervention

**DOI:** 10.1007/s11033-020-05469-5

**Published:** 2020-05-06

**Authors:** Fabio Rossi, Martin Braun, Jonathan Brock, Janette Sen-Lum, Sonya Ellens, Elliot Lander, Douglas Stoddard, Carolyn Cross, Angelika Vance, Duncan Stewart, Bernard Thebaud, Judy Illes

**Affiliations:** 1 University of British Columbia, Vancouver, BC Canada; 2 Vancouver Laser and Skin Care Inc., Vancouver, BC Canada; 3 Cell Surgical Network, Rancho Mirage, CA USA; 4 SEMI, Toronto, ON Canada; 5 Ondine Biomedical Inc., Vancouver, BC Canada; 6 Ottawa Hospital Research Institute, Ottawa, ON Canada

**Keywords:** Ethics, Clinical, Health policy, Medicine and the law, Stem cell therapies

## Abstract

Despite years of warnings by the academic community that for most of the stem cell-based therapies offered in the private arena little evidence of efficacy exists, these services have been increasingly offered by Canadian private clinics. Recently, as the culmination of years of clashes between stem cell researchers and therapy providers, Health Canada issued a statement prohibiting any type of cell therapy that is not specifically approved. In this climate of conflict, a small group representing both these communities as well as the government gathered in Vancouver to identify common values, and agree on principles to move forward constructively. This historic moment demonstrated that even in this contentious space a meeting-of-minds in between researchers, clinicians, ethicists, entrepreneurs and other stakeholders is possible.

The boundary between hope and the hype generated by advances in the stem cell field is neither easy to draw nor define. On one side of the boundary, the potential of stem cells is enormous: a large number of studies point to the possible benefits of harvesting “stem cells” from an anatomical location, often subcutaneous adipose, and transferring them to another location affected by disease, usually involving inflammation [1, 2]. On the other, realizing that potential in practice will still require additional years of clinical testing and implementation. Studies are not yet sufficiently powered to be convincing on their own, and while meta-analyses in aggregate suggest safety, they have also failed to give clear indications of whether these procedures work or not [3, 4]. Nonetheless, hope and promise have prompted the growth of for profit clinics providing “stem cell treatments” [5, 6]. In parallel, the associated hype has become a central concern of a watchful academic world, prompting accusations of charlatanism and exploitation of people with chronic health conditions (7–9). This tension has recently escalated as Health Canada issued a stern warning that any autologous therapy performed with “harvested and manipulated” cells is considered a drug and as such needs approval (https://healthycanadians.gc.ca/recall-alert-rappel-avis/hc-sc/2019/69974a-eng.php).

Indeed, private sector providers of stem cell interventions and stem cell researchers have been at loggerheads for more than a decade [10]. The first direct-to-consumer purveyors of cell-based interventions opened at the beginning of the millennium in a number of international locations characterized by relaxed regulations. The trend gave rise to the phenomenon of “stem cell tourism,” and created concerns that the Canadian health system would have to provide follow-up care for the potential side effects of unapproved therapies received abroad. Against the backdrop of criticism, providers dug in their heels ever deeper, and academics, professional organizations and regulatory agencies including the Stem Cell Network (SCN), the International Society for Stem Cell Research (ISSCR), the California Initiative for Regenerative Medicine (CIRM) and the Food and Drugs Administration (FDA) escalated their reactions with active guidance for the public and patients. Still, the public fueled demand [5]. Large numbers of patients have pursued these treatments through out-of-pocket payments. Well over 100 patients per month have been injected with adipose derived cells in recent years, mostly into inflamed joints, in the context of such “patient-funded experimentation”. Until recently Health Canada struggled with questions about whether the transfer of unmanipulated cells from an anatomical location to another is medicine or surgery and, as such, not under Canadian regulatory control. Other autologous transplants such as those of bone, skin and ligaments are currently not considered drugs. Indeed, even according to the most recent Health Canada statement, the use of lymphohematopoietic cells that are contained at variable frequencies in all cell preparations are allowed in an attempt to avoid interfering with routinely used procedures such as hematopoietic stem cell transplants.

This hyperpolarized impasse is fundamentally harmful to patients and untenable. In an attempt to explore and possibly reconcile differences between the two groups, a meeting of stakeholders and regulators was organized at the University of British Columbia under the auspices of the BC Regenerative Medicine Initiative and of Neuroethics Canada. In this context researchers, clinicians, ethicists, entrepreneurs, government and other stakeholders came together to create a bridge in this contentious space.

We identified seven principles (Box 1) that focus on clinical need, differentiation of disease categories, and the balance between innovation and regulation.

**Box 1:** Suggested principles when considering regulation of stem cell treatments

First principles
∙ Stem cells hold tremendous promise.
∙ There is a significant unmet clinical need for discoveries across a range of health conditions (e.g., neurologic, autoimmune, inflammatory).
∙ Different areas of stem cell clinical interventions/cell therapies should be separated out.
∙ Interventions must be delivered by qualified practitioners only.
∙ Different models of doing business to incentivize participation and advancement of the field must be respected.
∙ Regulatory considerations, health and safety and public trust, are paramount.

The interpretation of the core values, such as “first, do no harm” and “patient best interests”, will vary by provider and by patient, be subject to influences, and represents a balance between clinical judgement and patient preferences and perspectives. We agreed that there is a role for compassionate use in some cases. Context matters.

We agreed that patient safety is an overriding priority, and that it dictates important limitations to what interventions should be delivered and in which settings. For example, unmanipulated cells from autologous adult sources are generally safe, as long as they are processed appropriately and delivered by trained practitioners. By contrast, cells from embryonic or fetal sources carry a real risk of uncontrolled proliferation and possibly malignant transformation, and should only be used in carefully controlled and regulated clinical trials [11, 12].

The clinical use of stem cells outside of a clinical trial should also be backed by a significant body of literature supporting effectiveness and safety. For example, the evidence that adipose-derived cells may be beneficial in neurodevelopmental conditions is essentially negligible, but a substantial body of literature suggests that they may have anti-inflammatory properties, and legitimate clinical trials are being conducted based on this premise (13). Therefore, while offering these interventions for diseases with a strong inflammatory component (e.g., osteoarthritis) is backed by rationale, offering them to cure autism is just preying on desperate hopes.

We failed to find an agreement on matters pertaining to access and distributive justice. Tensions of conflict of interest arise when medical and financial needs clash.

We were also left with a number of other vexing challenges: For example, why does this space seem messier, and why does it receive more scrutiny than others in medicine? Is it more unethical to offer a stem cell treatment or a lift of the buttocks in which similar lipoaspirates are used, but injected in different anatomical locations for cosmetic reasons? How can peer review and needs of clinical environment be balanced? Who would regulate patient funded experimentation and monitor standardization, and what would constitute controls? Is there latitude for off-label work and where do patients lie on this landscape? These questions will only be answered productively if stakeholders cooperate, which we initiated through this meeting, and continue to do so in good faith.

Precisely because it is difficult to arrive at a firm conclusion on efficacy based on the current literature, data are needed from the troves of patients treated with stem cell therapies who fall under the radar of the healthcare system today. We conclude, therefore, with a call for a Canadian Stem Cell Database to be established and an invitation for providers to participate, and offer strategies to achieve this goal. We propose:

- *Start small and laser-focused*: To begin, target specific applications, such as the highly represented musculoskeletal applications, to garner support and rapidly build a useable database within that area.
- *Provide support to gather patient consent*: Critical to the growth and strength of the database will be patients’ informed consent to share their treatment and follow-up data for research purposes. Consent must be top-of-mind; strategies for inviting it must be both efficient while conforming to best practices in research ethics.
- *Consider what health data linkages will be valuable*: To be able to answer questions such as whether patients treated with stem cell interventions use related services such as surgeries and pain-control medications at the same rate as non-treated controls, the database must have the ability to track the progression of the conditions being treated. This can be achieved ideally by following patients’ trajectories through the health care system, and collecting and linking the relevant data.
- *Identify appropriate control populations*: A baseline healthcare system trajectory is needed so that questions about whether recipients of stem cell treatment deviate from it can be tested. Ideal control groups comprise large numbers of patients with a similar diagnosis (e.g., knee osteoarthritis). The implementation of this step will require engaged health practitioners who treat these patients and obtain consent, as described earlier, to collect and share treatment and health data.
- *Consider how to incentivize participation*: Private providers may require incentives to collect and make available data and samples to researchers from the control patients. We acknowledge that such endeavours come with a price tag and ethical responsibilities that may discourage participation. However, we suggest that a database forms the basis of a validation program that recognizes players in the field—essentially, an identifiable seal of participation and of willingness to play by the rules. While there is no guarantee that an intervention will work, participants will at least be recognized for being among the group of innovators and entrepreneurs doing their part to assess if it does. They will have access to prescribed methods for qualitative data collection, schemes for quantitative data pre and post intervention, and potentially a pool of statistical consultants and methodologists, information technologists and updates.

We appreciate the criticism we have seen through social media about “garbage [data] in; garbage out.” We resist this criticism with the belief that action is better than the *status quo*, and that some data is better than no data. With an unbreakable impasse as the historical alternative, we feel that benefits of this experiment outweigh its risks.

To conclude, we assert that each stakeholder has a key role to play. The continued active involvement of Health Canada is essential to enable the shared vision, goals and methods we propose here. Oversight is important, but self-regulation in the attitudes and practice of researchers and providers is far more strategic and efficient. Patients must scrutinize the clinics they consider for therapy, ensure the clinics are playing by the rules to which they have expressly signed up, and fulfill test requirements and standardized reports of outcomes over time.
